# Data availability and usability of a remote device for vital sign monitoring on general wards and home: a single-center prospective, observational study

**DOI:** 10.1007/s10877-026-01448-6

**Published:** 2026-06-25

**Authors:** A. Jonkers, M. de Groot, M. Duijvestein, P. R. de Reuver, G. Huisman-de Waal, S.J.H. Bredie, I. M. Spenkelink

**Affiliations:** 1 Department of Surgery, Radboud University Medical Centre, Nijmegen, The Netherlands; 2 Department of Gastroenterology and Hepatology, Radboud University Medical Center, Nijmegen, 6525 GA The Netherlands; 3 Health Innovation Labs, Radboud University Medical Centre, Nijmegen, The Netherlands; 4 Department of Internal Medicine, Radboud University Medical Centre, Nijmegen, The Netherlands

**Keywords:** viQtor, ViSi Mobile, Device, Continuous monitoring, General ward, Hospital

## Abstract

Continuous monitoring with wireless wearable devices enables early detection of clinical deterioration and supports post-discharge monitoring. This study evaluated data availability and patient usability of a novel upper-arm wearable sensor for continuous monitoring, on the general ward and at home, and compared its intramural performance with the current local standard. This prospective observational study was conducted at an academic hospital involving surgical and gastroenterological patients. The viQtor was used alongside ViSi Mobile during hospital admission and subsequently stand-alone for seven days post-discharge. Data availability was measured by analyzing the proportion of missing data, usability with the System Usability Scale (SUS), and data agreement by comparing in-hospital vital sign measurements between the viQtor and ViSi Mobile. Forty-five patients (median age 64 years) participated, of whom 35 (78%) continued using viQtor at home. Data gaps increased with patient activity during admission and at home. In-hospital data availability for the viQtor was similar for pulse rate (87% vs. 85%) but lower for respiratory rate (56% vs. 83%) and oxygen saturation (SpO₂) (48% vs. 84%) compared to ViSi Mobile. At home, the data availability of the viQtor for pulse rate, respiratory rate and SpO_2_ was 77%, 46%, and 39%, respectively. ViQtor achieved a mean SUS score of 71.1, indicating acceptable usability. ViQtor showed slightly higher pulse rate values, and lower respiratory rate and SpO₂ values compared to ViSi Mobile. Data availability of the viQtor was high for pulse rate but limited for respiratory rate and SpO₂, compared to the ViSi Mobile. Activity lowered data availability of all parameters, particularly at home. Patient usability was acceptable. The viQtor showed small but significant vital-sign differences compared with the ViSi Mobile. Reducing activity-related data loss may further improve applicability for continuous monitoring.

## Introduction

On general hospital wards, vital signs are typically assessed through intermittent nurse-performed spot checks, at least every 8 h, leaving patients unmonitored for most of their stay. As a result, early signs of deterioration frequently go unnoticed. Prolonged hypoxemia occurs in up to 90% of patients after non-cardiac surgery and often remains undetected [1]. Postoperative hypotension (mean arterial pressure < 65 mmHg for ≥ 15 min) affects approximately one fifth of abdominal surgery patients, yet is missed in nearly half of cases when relying solely on intermittent measurements [1]. Similar unnoticed periods exist for tachycardia, bradycardia, tachypnea, and bradypnea [2]. 

Such unrecognized events delay critical interventions and frequently precede further escalation of care and unexpected ICU transfer [3, 4]. Nearly half of all in-hospital adverse events occur on general wards, and the European Surgical Outcomes Study (EuSOS) showed that three quarters of surgical patients who died in hospital were never admitted to the ICU, underscoring the pivotal role of adequate ward monitoring in preventing postoperative deterioration and life-threatening complications [5, 6]. 

ViSi Mobile™ (Sotera Wireless, San Diego, CA, USA) is a continuous, non-invasive monitoring system that enables early detection of subtle vital changes, by integrating real-time data into patients Electronis Healthcare Record to support timely intervention, reduce ICU admissions [7–10], and improve safety [11–16]. Despite these advantages, prolonged use of the ViSi Mobile has been associated with discomfort, skin irritation, and pressure ulcers, particularly in patients requiring extended monitoring or those with sensitive skin [17, 18]. In addition, its setup and the volume of continuous data can be time-consuming for nurses.

Recent technological advances have enabled smaller, wireless, and more skin-friendly alternatives. One such device is the viQtor™ (smartQare, Eindhoven, The Netherlands), a compact upper-arm system designed without wires or adhesive patches to improve patient comfort and mobility. Although the viQtor measures fewer parameters than the ViSi Mobile, its ease of use and minimal handling may reduce the nursing workload and support broader implementation, including at a home setting [7, 8]. 

This evaluation study had a threefold aim. First, assess feasibility of viQtor by evaluating data availability of the novel wearable device on general wards and at home. Second, assess the usability of viQtor from a patient perspective by evaluating user experience. Finally, to evaluate whether data from both devices are in agreement, the data obtained with viQtor was compared to data obtained with the ViSi Mobile.

## Methods

### Study design

To evaluate viQtor for both clinical and at-home use, a prospective observational study was conducted (June 2024- December 2024) at the departments of gastroenterology and surgery at Radboudumc, a large academic hospital in the Netherlands. This study reports on the use of viQtor in direct patient care where patients routinely are connected to a Visi Mobile device for continuous vital sign monitoring, followed by continued viQtor monitoring at home.

### Study outcome measures

The primary outcome measure was data availability of the viQtor device during hospital admission and home monitoring. Data availability was determined by the proportion of missing data per vital sign parameter. The in-hospital recorded data was compared to the data availability of the ViSi Mobile. Both the frequency and duration of data gaps were examined for both devices during overlapping monitoring periods. Only gaps > 1 min were analyzed, as the normal frequency of measurements for both devices is 1 min.

The second outcome measure was patient usability, assessed using the System Usability Scale (SUS) questionnaire [9, 10]. The SUS is a validated and widely used ten-item questionnaire that provides a global measure of perceived usability of medical devices and e-health applications. We used the Dutch version of the SUS, which has demonstrated good reliability and validity (Cronbach’s alpha 0.74). Items are scored on a five-point Likert scale ranging from 1 (strongly disagree) to 5 (strongly agree) and capture subjective perceptions of usability, including complexity, support, and training. Participants were instructed to provide immediate responses to minimize reflection time.

The third outcome measure was data agreement, assessed by the comparison between the viQtor and the ViSi Mobile. Agreement was evaluated based on per-patient comparison of vital sign values (pulse rate (PR), respiratory rate (RR), and oxygen saturation (SpO₂)) obtained from both devices.

### ViQtor

The viQtor (smartQare, Eindhoven, The Netherlands) is an upper-arm wearable sensor that uses high-frequency photoplethysmography (PPG) signal to derive PR, RR, and SpO_2_ at a minute-level resolution. It also tracks physical activity and skin temperature. Software version 2.0.1 was used during this study.

### Visi mobile

ViSi Mobile™ (Sotera Wireless, San Diego, CA, USA) is a continuous, non-invasive monitoring system that enables early detection of subtle vital changes. Since 2017, ViSi Mobile™ has been implemented at Radboudumc on eight general wards comprising 120 beds and serves as a benchmark for continuous monitoring. As part of standard care, all admitted patients were connected to a ViSi Mobile device. Therefore, PR, RR, SpO₂ and cuffless blood pressure were continuously measured with a complete set of these vitals registered to the Radboudumc database every minute. Monitoring with the ViSi Mobile was only performed within the hospital.

### Participants

Patients were recruited from surgical and gastrointestinal (GI) wards and selected based on the in- and exclusion criteria as shown in Table 1. Ethical approval was granted by the Radboudumc ethics committee (case number: CMO 2024–17141).

**Table 1:** In- and exclusion criteria

Inclusion criteria	Exclusion criteria
≥ 18 years	History of: - Cardiac arrhythmia - Tremors - Cardiopulmonary bypass - Allergy to metal or plastic - Edema - Upper arm deformities - Skin irritation - Degenerative changes - Contraindications for blood pressure cuffs - Limited blood flow
Connection to the ViSi Mobile system	Cardiovascular devices: - Implanted cardiac pacemaker - Internal cardioverter defibrillator - Chronic resynchronization therapy device
Scheduled for discharge within 48 h	Patients admitted for urgency surgery within 24 h from admission
Provision of informed consent	Patients at risk of vascular compromise of the arm on which device would be placed - Upper extremity deep venous thrombosis - Peripherally inserted central catheters - Radial arterial lines - Dialyses fistulas - Severe upper extremity trauma

For all participants, demographic data and clinical information, including underlying disease, length of hospital stay, and vital parameters, were collected.

### Study procedures

After providing informed consent, patients who were already routinely connected to continuous monitoring with the ViSi Mobile, were also connected to viQtor and received instructions on troubleshooting and battery charging of the latter device. Participants were instructed to wear viQtor continuously, removing it only for showering or charging. Following discharge, participants wore the device at home for seven days and recorded their daily activities in a diary, after which the data were assessed for availability and quality by the research team. Activity categories are listed in Table 9 (Appendix).

**Table 2:** Patient characteristics

Characteristics	Total cohort (*n* = 45)
Age y, [IQR]	64.0 [52.5–70.5]
Female sex n, (%)	11 (24.4%)
BMI, kg/m^2^ [median]	25.5 [22.6–28.1]
Reason of admission n, (%) Pancreatitis Cholangitis Decompensated liver cirrhosis Ileus GI bleeding GI surgery (oncology) GI surgery	11 (24.4) 3 (6.7) 4 (8.9) 6 (13.3) 5 (11.1) 4 (8.9) 12 (35.6)
Duration of admission days, median [IQR] Duration of wearing viQtor during admission days, median [IQR] Number of patients who took viQtor home, n (%) Duration of wearing viQtor at home days, median [IQR] Completed diaries (%)	5 [3–7] 2 [2–3] 35 (78) 7 [6–9] 28 (80)

BMI: body mass index, GI: Gastrointestinal

On day three, a researcher contacted each participant by phone to discuss their experience, answer questions, and remind them to charge the device. On the final study day, participants completed the SUS questionnaire about their experiences.

During the study, researchers and clinicians did not have access to viQtor’s real-time vital sign data, and viQtor data were not used for clinical decision-making. Clinical decisions during admission were based on the ViSi Mobile data as part of standard care in our hospital.

### Data processing and quality control

Activity levels detected by the viQtor were used as a proxy for wear time, as direct measures of sensor attachment were unavailable in this stand-alone study design. These activity levels range from 0 (no movement) to 5 (very active), providing an indication of patient mobility and physical engagement. To reduce misclassification of non-wear time as missing data, only periods with a detected activity score were included in the primary analysis; activity level 0 was considered valid and indicated that the patient was wearing the device but not moving. In addition, periods that patients recorded in their diaries as non-wear, coded as activity level 9, were excluded from the analysis to minimize inclusion of data from periods with detected activity resulting in false vital sign measurements. Data availability was calculated for each vital sign parameter as the proportion of valid measurements within the included periods.

Diary-reported activity levels and viQtor-derived activity levels were used to stratify data in groups to evaluate if activity influenced data availability.

To assess data agreement between viQtor and ViSi Mobile, data streams from both devices were synchronized on a one-minute timestamp and restricted to overlapping monitoring periods. Missing data were defined as absent values per vital sign during valid wear periods.

### Statistical analysis

Statistical analysis was performed using IBM SPSS 25 (SPSS Inc., Chicago, IL, USA).

Given the skewed distribution of key outcomes (e.g., data gaps), results are primarily presented as median (IQR). Mean ± SD is only reported for approximately normally distributed variables.

Continuous data were analyzed using Python (version 3.11). Data streams from the viQtor and the ViSi Mobile were synchronized to detect transmission gaps, and analyze agreement in vital sign measurements (PR, RR, and SpO₂). Bland–Altman analyses were performed using a repeated-measures approach to assess measurement bias and 95% limits of agreement (LOA) between the two systems, accounting for within-subject correlation by applying mixed-effects modelling with a random intercept for patient.

To calculate the SUS score, the score contributions were summed for each item (0–4). For questions 1,3,5,7, and 9 the score contribution is the scale position minus 1. For questions 2,4,6,8 and 10, the contribution is 5 minus the scale position. The total sum was multiplied by 2.5 for the overall SUS score and range from 0 to 100. Scores of 0–50 indicated ‘not acceptable’, 51–67 indicated marginal level, and 68–100 indicated ‘acceptable’ levels of usability [11]. 

Analysis of differences between both devices was performed using mixed linear model regression analyses. In cases where differences were not normally distributed, Wilcoxon signed-rank tests were applied. For the regression analyses, results are reported with a 95% confidence interval (CI); for the Wilcoxon tests, only p-values are presented.

## Results

### Patient characteristics

A total of 45 patients participated in this study, with a median age of 64 years (IQR 52.5–70.5). The cohort included more males (76%) than females (24%). The most common reasons for admission were GI surgery (36%) and pancreatitis (24%), followed by ileus, GI bleeding, decompensated liver cirrhosis, and cholangitis.

The median length of hospital stay was five days (IQR 3–7), during which patients wore the viQtor device for a median of two days (IQR 2–3). Overall, patients wore the viQtor for a mean duration of 8.9 days (12,826 min). A total of 38 of 45 participants (84%) completed the questionnaires; reasons for non-completion were not reported.

Overall, 38 of 45 participants (84%) completed the questionnaires; reasons for non-completion were not documented. After discharge, 35 patients (78%) continued viQtor monitoring at home for a median of seven days (IQR 6–9), of whom 28 (80%) completed the home diary. Patients who did not continue viQtor use at home reported that they did not feel able to test an additional device during their recovery. Table 2 summarizes the baseline characteristics of the study population.

**Table 3:** Overview questions System Usability Score with results

Question	Score (median, IQR)
I think that I would like to use this system frequently.	4 (3–4)
I found the system unnecessarily complex.	1 (1–2)
I thought the system was easy to use.	4 (4–5)
I think that I would need the support of a technical person to be able to use this system.	1 (1–2)
I found the various functions in this system were well integrated.	3 (3–5)
I thought there was too much inconsistency in this system.	1 (1–2)
I would imagine that most people would learn to use this system very quickly.	5 (4–5)
I found the system very cumbersome to use.	1 (1–2)
I felt very confident using the system.	4 (3–5)
I needed to learn a lot of things before I could get going with this system.	1 (1–1)

### Primary outcomes

#### Data availability of the viQtor

More data was missing during out-of-hospital use than during in-hospital use for all three vital sign parameters (Table 4). In hospital, PR was available for 87% of the time, RR for 56%, and SpO₂ for 48% of the time. During home monitoring, data availability decreased to 77% for PR, 46% for RR, and 39% of the time for SpO₂.

**Table 4:** Average data availability per patient for viQtor per vital parameter with viQtor-reported activity

In-hospital (*n* = 45)	Wearing minutes (average)	Pulse rate data points (percentage available)	Respiratory rate data points (percentage available)	SpO_2_ data points (percentage available)
	3017	2620 (87%)	1683 (56%)	1457 (48%)
Out-of hospital (*n* = 37)	8324	6437 (77%)	3797 (46%)	3264 (39%)

SpO_2_: Oxygen saturation, n: number of patients

During hospital admission, patients wore the viQtor for an average of 3,017 min, during which the device recorded 2,620 PR data points per patient (87% availability), comparable to the ViSi Mobile (2,438 data points; 81%). RR availability was lower, with 1,683 data points (56%), compared to 2438 (81%) of ViSi Mobile. SpO_2_ showed the lowest completeness, with 1,457 data points (48%), while the ViSi Mobile showed an availability of 83%. These findings indicate that the viQtor collected fewer data points overall, particularly for RR and SpO₂, whereas PR availability was relatively close to the ViSi Mobile (Table 5).

**Table 5:** Average number of data points per patient during admission for viQtor and ViSi Mobile, including percentage availability per parameter

Device	Timestamps (minutes with activity level)	Average minutes with at least one vital sign	Pulse rate available (%)	Respiratory rate available (%)	SpO₂ available (%)
viQtor	3017 (reference)	2625	2620 (87%)	1683 (56%)	1457 (48%)
ViSi Mobile		2645	2438 (81%)	2511 (83%)	2499 (83%)

SpO₂ = oxygen saturation

Patient-reported activity levels revealed that data availability at home was associated with patients’ mobility. During periods of rest (activity code 0), data availability was highest (PR 75.5%, RR 55.8%, SpO₂ 51.8%). When patients were active (activity codes 1–3), availability decreased substantially for RR (28.0–53.9%) and SpO₂ (20.6–52.3%) (Table 6).

**Table 6:** Availability of data per activity level as reported in diary

Activity code diary	Duration activity	Percentage pulse rate	Percentage respiratory rate	Percentage SpO_2_
0	29,677	75.5	55.8	51.8
1	18,051	59.1	28.0	20.6
2	15,948	64.5	35.7	32.6
3	1536	85.7	53.9	52.3
9	815	1.3	0.4	0.2

SpO_2_: Oxygen saturation, 0 = no activity, 1 = light activity, 2 = moderate activity, 3 = intense activity, 9 = patient reported not wearing

During in-hospital monitoring, the mean of maximum gaps per patient differed between devices across vital parameters (Table 7). For PR, the mean maximum gap was 35.6 min for viQtor and 41.9 min for ViSi Mobile. For RR, the mean maximum gap was 83.2 min for viQtor compared with 75.0 min for ViSi Mobile. For SpO₂, the mean maximum gap was 98.1 min for viQtor and 39.7 min for ViSi Mobile.

**Table 7:** Data gaps per vital sign for viQtor and ViSi Mobile at datapoints were viQtor recorded activity

Device	Vital sign	No. of gaps	Median gap (min)	Q1	Q3	Min gap (min)	Max gap (min)	Mean of max gap per patient (min)
viQtor (in hospital)	PR	14,245	1.0	1.0	1.0	1.0	423.0	35.6
viQtor (in hospital)	RR	30,997	1.0	1.0	2.0	1.0	426.0	83.2
viQtor (in hospital)	SpO₂	28,006	1.0	1.0	3.0	1.0	428.0	98.1
viQtor (at home)	PR	16,697	1.0	1.0	2.0	1.0	434.0	86.8
viQtor (at home)	RR	33,507	1.0	1.0	2.0	1.0	1,158.0	294.4
viQtor (at home)	SpO₂	29,936	1.0	1.0	2.0	1.0	1,158.0	310.0
ViSi Mobile	PR	5263	1.0	1.0	2.0	1.0	304.0	42.0
ViSi Mobile	RR	2256	1.0	1.0	2.0	1.0	545.0	75.0
ViSi Mobile	SpO₂	3515	1.0	1.0	2.0	1.0	296.0	39.7

Legend: Q1, Q3 = first and third quartile; PR = pulse rate; RR = respiratory rate; SpO₂ = oxygen saturation;

*Patient User Experience*.

The mean SUS score was 71.1 (IQR 64.4–80.6), indicating good overall usability. Table 3 provides an overview of item-level responses. Of the respondents, 7.9% had a SUS score < 50 and 18.4% scored between 50 and 67, while 73.7% scored > 67, indicating generally favorable usability perceptions. Participants reported that the system was easy to use (median 4, IQR 4–5), well integrated (median 3, IQR 3–5), and inspired confidence during use (median 4, IQR 3–5). Perceived complexity and inconsistency were rated low throughout (median 1 for both), and users indicated that little prior learning was required (median 1, IQR 1–1). Moreover, most participants believed that people would learn to use the system quickly (median 5, IQR 4–5), while the need for technical support was considered minimal (median 1, IQR 1–2).

**Table 8:** Median differences (IQR) between viQtor and ViSi Mobile for pulse rate, respiratory rate, and oxygen saturation

Parameter	Difference (Median, IQR)	*p*-value
Pulse rate (bpm)	0.79 (− 10.60–12.17)	< 0.001
Respiratory rate (/min)	−1.35 (− 9.69–6.98)	< 0.001
Oxygen saturation (%)	−1.18 (− 7.17–4.81)	< 0.001

p-values from Wilcoxon signed-rank tests

#### Data agreement

**Fig. 1:**
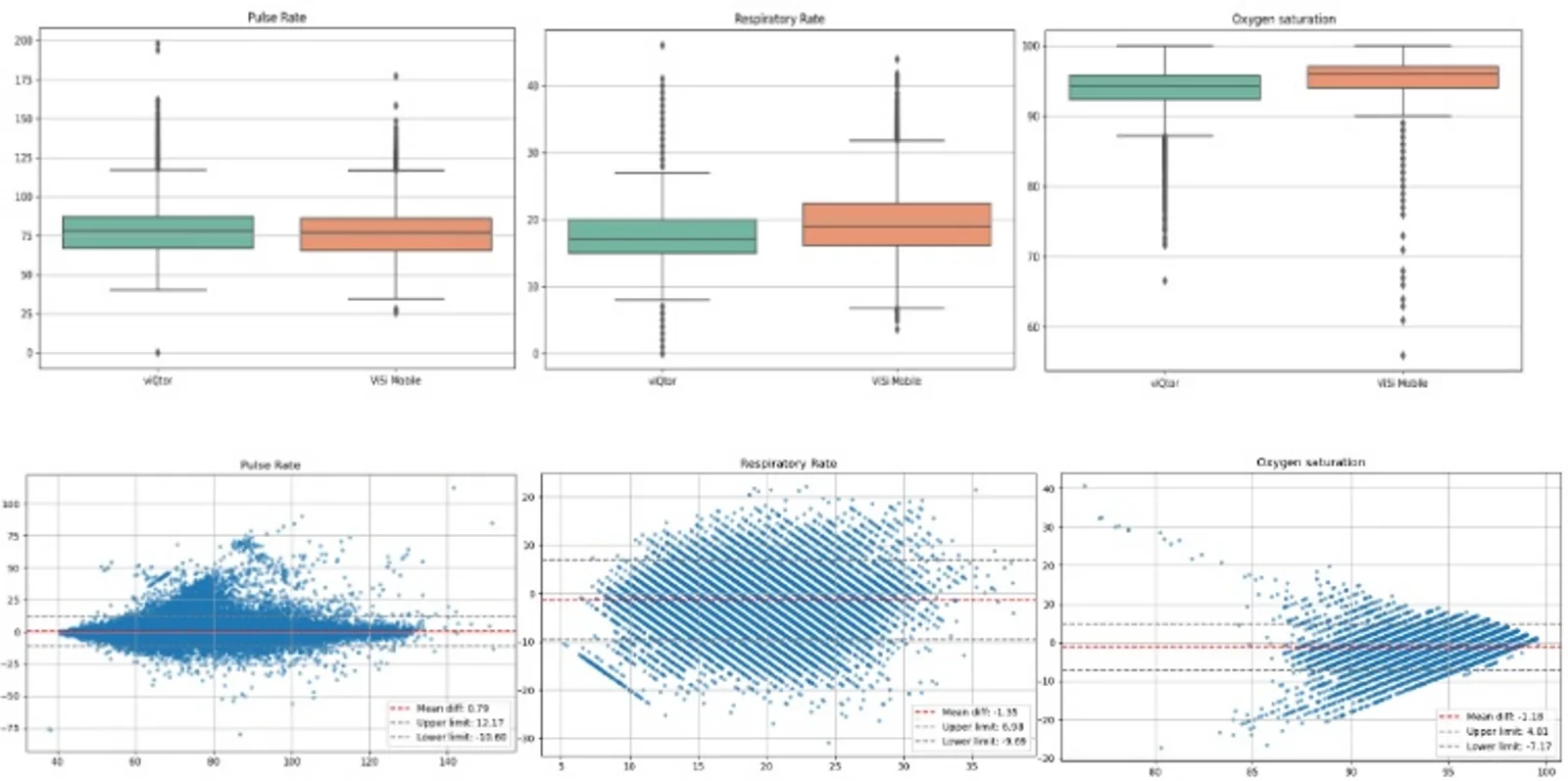
Comparison of measurements between viQtor and ViSi Mobile. The upper row shows boxplots of pulse rate (equivalent to pulse rate), respiratory rate, and oxygen saturation obtained from both devices. The lower row shows Bland–Altman plots, where the mean of the two devices (x-axis) is plotted against their difference (y-axis; viQtor – ViSi Mobile). A positive difference indicates that viQtor measured higher values than ViSi Mobile, while a negative difference indicates lower values. Red dashed lines indicate the mean difference (bias), and black dashed lines represent the limits of agreement

Figure 1 compares PR, RR, and SpO_2_ measured by the viQtor and the ViSi Mobile. The boxplots in the upper row illustrate the ranges and distributions of the measurements for each parameter, showing overall similar variability across subjects. The viQtor tended to report slightly higher RR and marginally lower SpO_2_ values compared to the ViSi Mobile.

The Bland–Altman analyses indicate overall agreement between the devices. For PR, the mean difference was minimal (0.79 bpm, 95% LOA: −10.60, 12.17), indicating close correspondence. RR showed a bias of − 1.35 breaths/min (95% LOA − 9.69, 6.98 breaths/min) and SpO2 a bias of − 1.18% (95% LOA − 7.17, 4.81%), indicating that the viQtor tended to report slightly lower RR and SpO_2_ values compared to the ViSi Mobile. There were small but statistically significant differences between devices for all parameters, with no indication of substantial disagreement (*p* < 0.001, Table 8).

**Table 9:** Activity codes diary

Activity code	Description	Activity
0	Rest	Sleeping
1	Light activity	Housework, shopping, etc.
2	Moderate activity	Gardening, walking, etc.
3	Heavy activity	Therapy, training, etc.
9	Not worn	

**Fig. 2:**
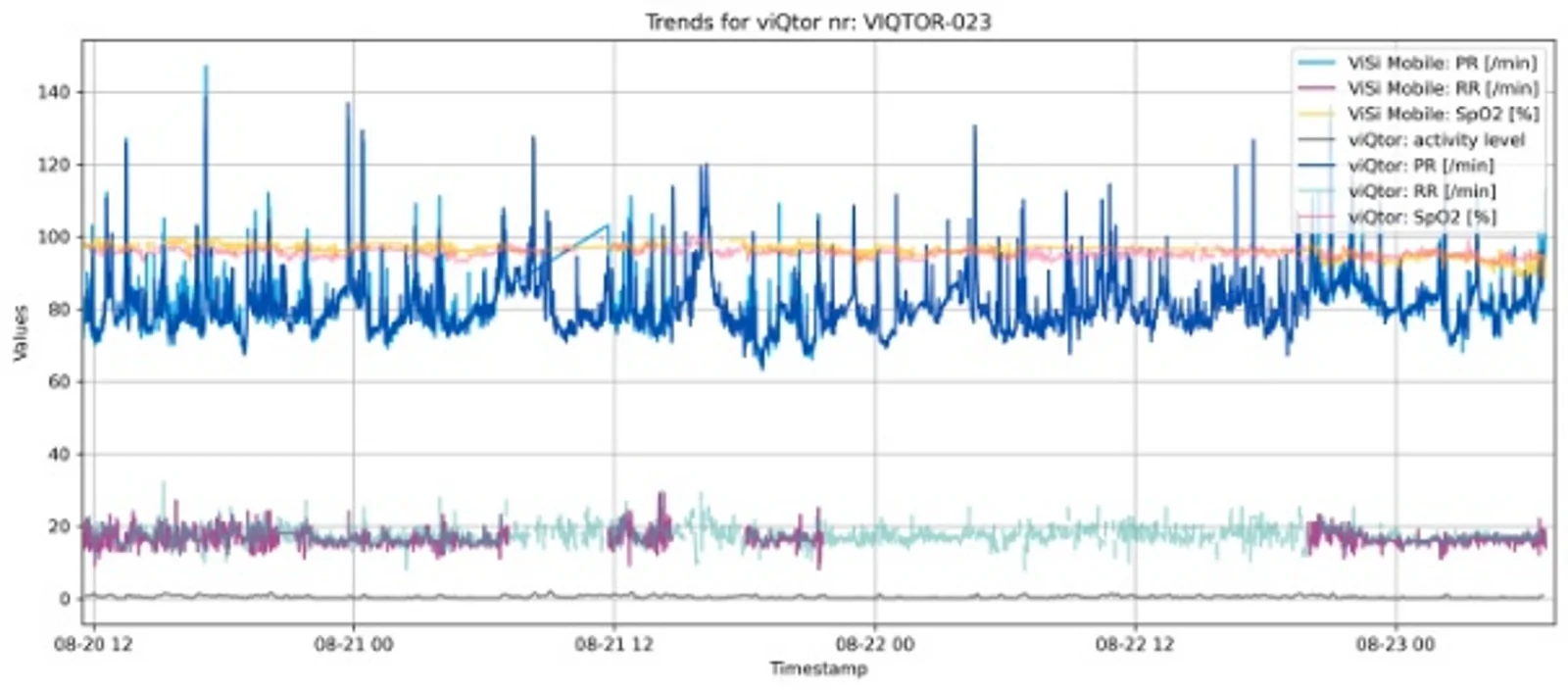
Overview of vital parameters of both devices in one of the subjects during hospital admission. The x-axis represents the timestamp of the data, with a clearer indication of time intervals. The graph includes values for various vital signs, with each parameter color-coded; PR: pulse rate, RR: respiratory rate, SpO2: Oxygen saturation

**Fig. 3:**
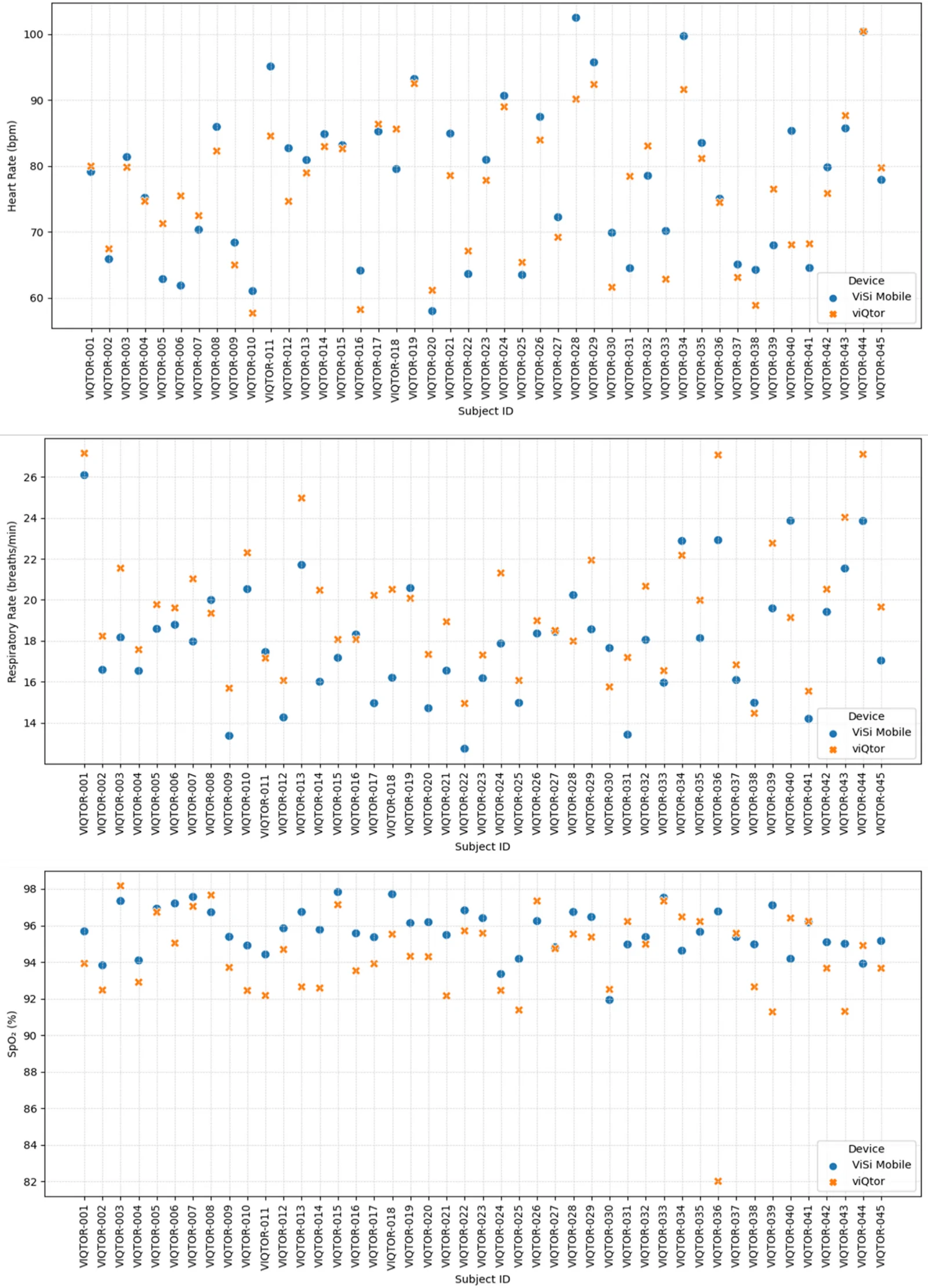
Mean values of pulse rate (top), respiratory rate (middle), and oxygen saturation (SpO₂) (bottom) per subject, measured by ViSi Mobile (blue circles) and viQtor (orange crosses). Each data point represents the average measurement for one subject, allowing direct comparison between the two devices across individuals

**Fig. 4:**
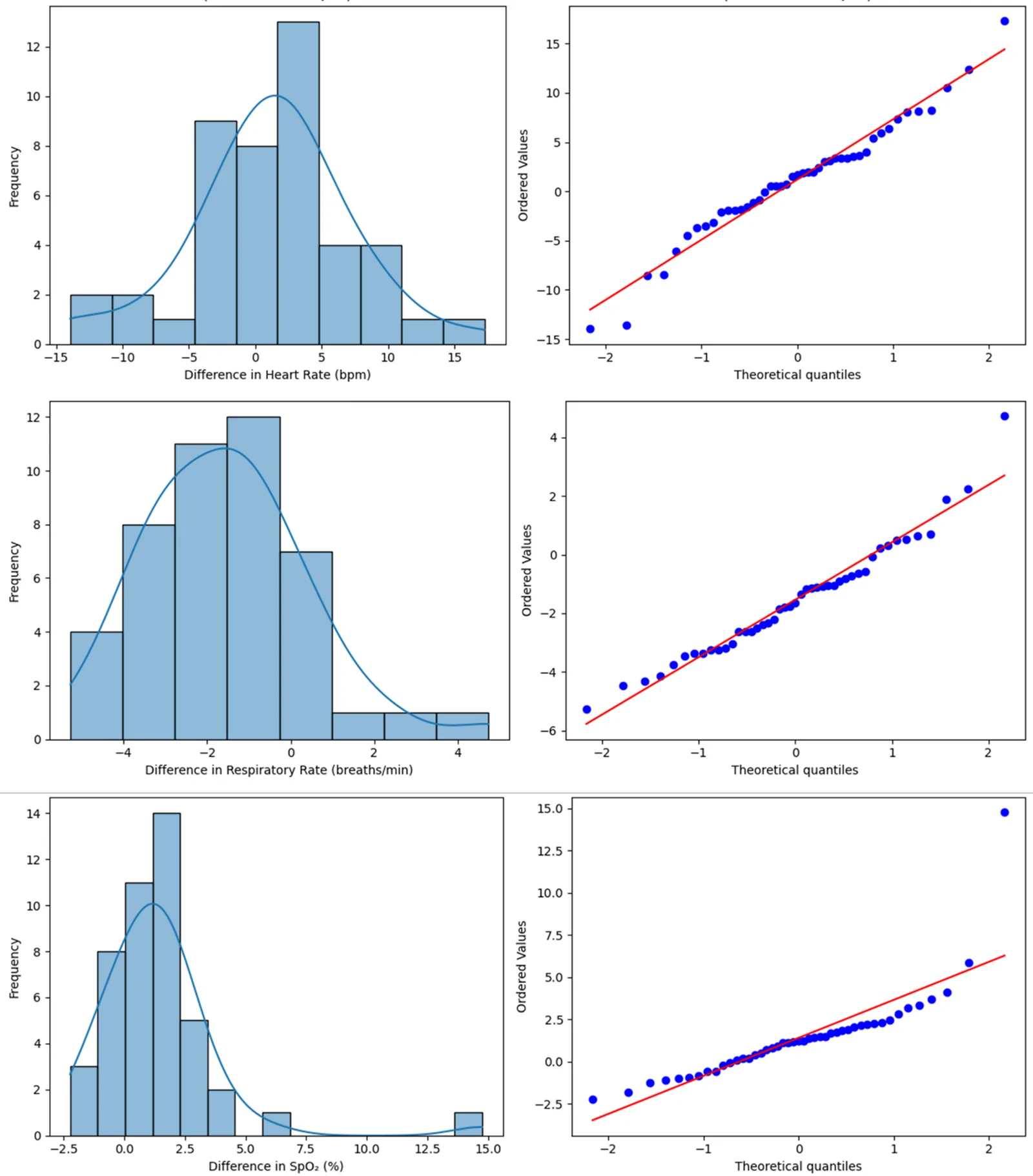
Distribution of measurement differences between ViSi Mobile and viQtor for pulse rate (top), respiratory rate (middle), and oxygen saturation (SpO₂) (bottom). Left panels show histograms of the differences with fitted normal curves, illustrating the spread and central tendency of the data. Right panels show Q–Q plots of the same differences, comparing observed values to the theoretical normal distribution to assess normality. Differences are expressed in bpm (pulse rate), breaths/min (respiratory rate), and percentage points (SpO₂)

Examples of device comparisons per subject, as well as histograms and Q-Q plots of the differences are displayed in Figs. 2, 3 and 4. Figure 2 displays a regular registration of both the viQtor and the ViSi Mobile in a sample person during admission to a nursing ward. The patient wore both devices from 2024-08-21–2024-08-23. Overall, the two devices show substantial overlap in PR and SpO_2_ trends, with values tracking closely over time. RR shows similar patterns, although viQtor measurements are more intermittent and show slightly greater variability.

## Discussion

This study evaluated the novel device viQtor for continuous vital sign monitoring during both hospitalization as well as after discharge at home. The main findings are that the viQtor has a good availability for pulse rate but limited availability for respiratory rate and SpO₂ and that user-friendliness and patient usability of the novel device are acceptable. Data availability in the hospital was good for PR (87%), but substantially lower for RR (56%) and SpO_2_ (48%). Availability declined even further in the home setting, with an additional 10% reduction associated with higher levels of patient activity. Compared with clinically derived measurements from the ViSi Mobile, the viQtor provided comparable availability for PR but significantly lower availability for RR and SpO₂.

The viQtor achieved a System Usability Scale (SUS) score of 71.1, reflecting acceptable user experience [9]. This level of usability may well be attributed to its smaller size and less invasive design, addressing known drawbacks of bulkier monitoring solutions like the ViSi Mobile. Given its compact design and absence of adhesive wires or electrodes, the viQtor may reduce, or even eliminate complications commonly seen with prolonged use of monitoring systems which are larger, heavier, and require adhesive patches, leading to discomfort, skin irritation, and pressure ulcers [12, 13]. Improved comfort and reduced invasiveness may enhance patient compliance, facilitating implementation in clinical settings, as higher usability scores have been linked to better adherence and integration of monitoring technologies in practice [14]. 

Although RR and SpO₂ availability of 56% and 48% respectively provide more frequent information than the standard intermittent nurse-performed spot checks, these levels remain below the current experience with continuous monitoring using a ViSi Mobile. Median gap durations were similar and both devices showed large maximum gaps. However the longer maximum gaps for SpO₂ observed with the viQtor indicate reduced signal continuity. Such prolonged gaps for SpO₂ information could delay recognition of patient deterioration and limit clinical usefulness [1, 15, 16]. The interruptions likely result from a combination of improper tightening of placement of the armband, sensor limitations, algorithmic artifact rejection in the PPG signal, and intermittent connectivity. The observed reduction in data availability during periods of increased patient activity is consistent with previous research and represents a known challenge in wearable continuous monitoring. In contrast, PR availability of 87% aligns well with expectations and current literature, suggesting robust performance for this specific parameter [17–19]. 

We observed small but statistically significant differences between the ViSi Mobile and the viQtor for PR, RR, and SpO₂. However, mixed-effects agreement analysis accounting for repeated measures and within-subject correlation showed wide 95% limits of agreement, particularly for SpO₂ (− 7.17 to 4.81) and pulse rate (− 10.60 to 12.17). This exceeds commonly accepted clinical margins of about ± 10 bpm for pulse rate, ± 2–3 breaths/min for respiratory rate, and ± 2–3% for SpO₂, for which narrow margins are especially important to detect hypoxemia [20, 21] This indicates that the two devices can produce clinically meaningful different values when measuring the same physiological signal. However, these findings should be interpreted in the context that ViSi Mobile is not a gold standard; the results reflect comparative agreement rather than validation against an absolute reference.

### Limitations and strengths

This study has several limitations to consider. First, the short monitoring period and the absence of real-time interpretation of the viQtor monitoring data for clinical observation during this study restrict both assessment of its ability to detect clinical deterioration and the generalizability of the findings to routine clinical practice. Consequently, it remains unclear whether the observed data availability and agreement, particularly for respiratory rate and oxygen saturation, are sufficient to support detection of clinically relevant events. Accordingly, no conclusions can be drawn regarding its impact on patient safety or clinical outcomes. Future studies should evaluate whether data completeness and signal continuity are adequate to reliably detect deterioration and support early intervention, including both trend monitoring and threshold-based decisions.

Second, although patient usability was assessed using the validated SUS, no equivalent usability assessment was performed for the ViSi Mobile. As a result, a direct comparison of user experience between the two devices is not possible, which limits conclusions about their relative acceptability and ease of use. Third, while we asked patients to record periods of not wearing the viQtor in their diaries, we do not have reliable information on the actual attachment times of both the viQtor and the ViSi Mobile. To address this for the viQtor, we used device-detected activity as a proxy for wear time which may have introduced misclassification. Since detected activity does not necessarily indicate correct sensor positioning, periods with device movement despite improper attachment may have been incorrectly classified as valid wear time, potentially biasing estimates of data availability. Additionally, no comparable correction was possible for the ViSi Mobile. This limitation is inherent to the stand-alone design, in which no independent verification of device attachment was available. These findings should therefore be interpreted with caution, and future studies incorporating direct measures of wear time or alternative definitions are warranted.

Finally, the user experience of the viQtor from the perspective of healthcare staff, an essential factor for successful clinical implementation, was not assessed [14]. This limitation can be explained by the stand-alone set-up for the purpose of this study, in which measured vital parameters could not be retrieved or displayed integrally in the EMR. Incorporating user feedback is therefore recommended and performed for future research [22]. Despite these limitations, this first study to evaluate the viQtor in both hospital and home settings provides valuable insights into data availability and patient experience through the use of the validated SUS. In addition, our comparison of this novel device with our current in-hospital standard broadens knowledge regarding alternative options for continuous monitoring.

### Future directions

Within this study, version 2.0.1 of the software was installed on the viQtor. SmartQare is currently working on further improving its algorithm to reduce gaps in RR and SpO₂ measurements, and showed enhanced data availability in PACU patients [8]. Therefore, the findings presented here reflect the performance of the device at the time of the study. Weather the new version also improved data availability at general clinical wards should be evaluated in future studies.

As continuous monitoring advances, new wireless blood pressure measurement options are emerging. Adding features like blood pressure and skin or core temperature measurements (already available with the ViSi Mobile) could enhance viQtor’s clinical usability and acceptance among nursing staff [19]. Meanwhile, it remains unclear whether measuring all vital signs every minute is clinically necessary, highlighting the importance of future studies aimed at identifying which monitoring frequencies are required, potentially specified for patient population, clinical wards and monitoring settings. Additionally, studies that compare more parameter-complete wearable devices with current systems, such as the ViSi Mobile, will provide valuable insights into their applicability and limitations.

Developing an algorithm that integrates vital parameters and generates an alert for clinical deterioration could facilitate broader use of CM. Evaluating the experiences of nursing staff is also advised, as their feedback is crucial for successful implementation and adoption of continuous monitoring [13, 23].

## Conclusion

In conclusion, the viQtor offers a promising solution for continuous patient monitoring in both clinical and at-home settings. Patient usability was acceptable, and the device demonstrated high data availability for PR, but limited for respiratory rate and SpO₂ in a real clinical environment. Therefore, further optimization is needed to minimize data loss, particularly due to patient activity in the home setting. As there is currently no consensus on the required vital parameters and acceptable data gaps, additional research is essential to ensure minimal requirements for data availability, data agreement and parameter relevance for continuous monitoring on general wards.

## Data Availability

Data is available on request.
